# Editorial

**Published:** 1841-03-27

**Authors:** 


THE MEDICAL EXAMINER.

PHILADELPHIA, MARCH 27, 1841.

### MEDICATED BATHS.

In a late number we alluded to a deficiency in our therapeutic means from the want of medicated baths adapted to general practice. Although many causes might be assigned for the neglect long attendant on this subject, so important to the health, especially of that class of citizens whose limited means debar the enjoyment of many comforts deemed necessary to the support of a sound constitution, we do not believe that any thing has exercised so injurious an influence as the apathy of professional men, and their indifference to, or perhaps ignorance of the extensive prophylactic and healing effects of such baths. Thus it has happened that empirics have obtained possession of this branch of therapeutics, so well appreciated and usefully applied of old, and as a natural consequence, the faculty have found themselves obliged either to approve the practice, and risk the charge of encouraging empiricism, or to condemn it altogether, and entirely deprive themselves of the privilege of using means so simple and yet so efficacious. If to this we add the supposed trouble and expense of such baths, we shall have given at least the most common difficulties heretofore in the way of their establishment in this country.*

In calling the attention of physicians to this subject, especially those connected with the hospitals, we do not design arguing the claims of vapour baths as powerful therapeutic agents. This may be done at another time; our object now is simply to demonstrate the practicability of their erection by showing the very small expense it involves. This may be briefly done by reference to the baths in the Hospital St. Louis, erected nearly thirty years since by D'Arcet. He tells us in his "Descriptions des Appareils à fumigations," that the cost of constructing an apparatus with twelve compartments for as many patients, may be valued at fifteen hundred francs; that of an apparatus for one patient at three hundred and fifty. In the large apparatus, the sulphur, or juniper berries, used at one bath for twelve persons, (assuming them as average samples of the mineral and vegetable substances employed,) cost a sum less than fifty sous, or about four and one-sixth sous for each person; to this must be added the expense of fuel, the cost of which was less than a sous to each patient. Supposing the average course for each case to be ten fumigations, the whole costs of the same amount to less than fifty sous. All of these estimates are as applicable here as in France, since the difference in the cost of the material used in vapours is more than compensated for by the diminished expense of fuel, which is nearly one-half lower in this country than in France.

Thus, at a cost of erection less than three hundred dollars, an apparatus may be constructed for twelve patients, or for seventy dollars one for a single patient, in which, for the trifling sum of less than *five cents* may be given the dry or moist (aqueous) vapour-bath, fumigations of alcohol, aromatic substances, &c., either moist or dry, mineral and vegetable fumigations, &c. &c.; they may be also made either local or general, a simple douche over a circumscribed spot, or to the whole surface of the body, at any temperature, and for any length of time.

As this statement is made upon the supposition that an establishment of this kind, if ever undertaken, must be the work of a public charitable institution, no estimate for buildings is included in it.

There would be no occasion for any such expense here, the two largest hospitals having abundant room within their walls for all purposes.

In the city a fair opportunity is now offered for an effort at accomplishing so desirable an end, a portion of the Pennsylvania Hospital has been lately vacated by the removal of the insane patients into the country. To what better and more useful purpose could one of the small outbuildings be devoted than to the erection of vapour-baths for the use of the hospital, and for patients in limited circumstances unconnected with the house, numbers of whom would be sent by their physicians to take the baths, for which a large proportion would pay a trifling sum, sufficient in the aggregate, it is believed, nearly to defray the current expenses of the establishment, while the better class of patients who would gladly avail themselves of the baths, would create an actual revenue by the higher prices they would pay for the same.

* In Staunton, a small town in Western Virginia, an establishment of this kind was erected by a Frenchman, a few years since, who offered, by means of the baths, to cure all "disorders of our weakly frames." We think he used, in addition to the baths, the medicine of Le Roy. Undoubtedly much good was occasionally done by the baths, and perhaps much more would have been effected, but for the unscientific mode of administering them. We remember to have heard that the owner spoke of dissolving *brimstone in cold water* as a "part of his system," to the deficiencies of which chiefly may be attributed his ultimate failure. He was last heard of with his baths at the Blue Sulphur Spring in Virginia.

We earnestly commend this subject to the consideration of those members of the profession whose connection with our charities brings them into frequent contact with the managers. They have it in their power to do much good by urging its claims, and unless by them it will most probably never be attended to.

